# A framework for early-stage sustainability assessment of innovation projects enabled by weighted sum multi-criteria decision analysis in the presence of uncertainty

**DOI:** 10.12688/openreseurope.18195.1

**Published:** 2024-08-05

**Authors:** John Henderson, Robert Peeling

**Affiliations:** 1 Colony, 5 Piccadilly Place, Britest Limited, Manchester, Greater Manchester, M1 3BR, UK

**Keywords:** Sustainable process design, Decision analysis, Decision processes, Multiple criteria analysis, Decision making under uncertainty, Manufacturing process scale-up risk management

## Abstract

A two-level hierarchical framework for early-stage sustainability assessment (FESSA) amongst a set of alternatives applicable from the earliest stages of process or product development is introduced, and its use in combination with an improved method weighted-sum method multi-criteria decision analysis (WSM-MCDA) in the presence of uncertainty is presented through application to a case study based upon a real-world decision scenario from speciality polymer manufacture. The approach taken addresses the challenge faced by those responsible for innovation management in the manufacturing process industries to make simultaneously timely and rational decisions early in the innovation cycle when knowledge gaps and uncertainty about the options tend to be at their highest. The Computed Uncertainty Range Evaluations (CURE) WSM-MCDA method provides better discrimination than the existing Multiple Attribute Range Evaluations (MARE) method without the computational burden of generating heuristic outcome distributions via Monte-Carlo simulation.

## Plain Language Summary

This paper introduces a framework that teams can use to think systematically about the wide range of criteria which go into deciding whether a proposed innovation enhances sustainability or not and shows how an improved method for multiple-criteria decision analysis can be used to put it into practice with an example drawn from the speciality chemicals industry.

Innovation in the manufacturing process industries requires decisions to be made. In individual projects, scientists and technical managers must decide which technology, materials, and equipment to use. Equally, those responsible for directing a portfolio of projects must choose which projects to prioritise. In either case, *early* decision making is desirable to avoid sinking time and money into dead-end projects, and to identify what further work is needed for projects with a future. The earlier you decide however, the harder it can be to obtain firm evidence (e.g. conclusive experimental data, fully validated costings, or life cycle impacts) upon which to base your decision. The growing societal expectation that sustainability criteria are factored into such decisions merely adds to the challenges faced by the decision maker.

Decisions must be made upon the evidence that *is* available combined with the informed judgement of those with knowledge of the system under consideration. This is best approached as a facilitated, team-based activity where assertions, assumptions and interpolations or extrapolations from the limited data can be tested and challenged. A sound decision-making process needs a suitable computational method for turning this complex qualitative and semi-quantitative assessment into a clear output indicator of potential success or failure for the options under consideration. The method described in this paper addresses this need but, just as importantly, the methodology ensures that the thought process behind whatever decision is indicated is clearly and transparently documented for future reference.

## Nomenclature

AHP                                        Analytic Hierarchy Process

CSTR                                      Continuous Stirred Tank Reactor

CURE                                     Computed Uncertainty Range Evaluations

ELECTRE, ELECTRE III     Elimination and Choice Translating Reality

FESSA                                    Framework for Early-Stage Sustainability Assessment

LCA                                       Life Cycle Assessment

MARE                                    Multi-Attribute Range Evaluations

MCDA                                  Multiple-Criteria Decision Analysis

MCDM                                   Multiple-Criteria Decision-Making

SAP                                      Super Absorbent Polymer

SURE                                   Simulated Uncertainty Range Evaluations

SSbD                                   Safe and Sustainable by Design

WSM                                   Weighted Sum Method 

## Introduction

### Constructing a framework for early-stage sustainability assessment

Selecting between manufacturing process options for the best alternative from a sustainability perspective is complicated by the competing issues at play. Guidance to facilitate systematic consideration of how well available routes address suitable criteria has the potential to help greatly with the decision-making process. We have therefore attempted to construct a standard but flexible framework of criteria targeted at early-stage process development. The Framework for Early-Stage Sustainability Assessment (FESSA), described in detail in the methods section, is designed for utility. It may be thought of as means of laying down the groundwork ahead of formal assessments such as Life Cycle Assessment (LCA) or social-LCA, at a point where gaps and uncertainties in hard data typically makes a complete formal assessment problematic.

The thinking behind the framework has been influenced by a variety of published approaches which variously systematise parts of the elements it brings together, such as risk assessment ^a^ ( Manipura *et al*., 2013), process flexibilities ^b, c^, environmental impacts/sustainability ^d^ ( Peace *et al*., 2018), and social sustainability ^e, f^, but it is also the product of evolutionary co-design in consultation with Britest members and collaborative partners as it has been applied to real business cases. 

### Multiple-Criteria Decision Analysis

Multiple-Criteria Decision Analysis (MCDA) enables those involved in managerial decision-making to take an explicit, structured approach to considering multiple criteria (or objectives) of the sort provided by the FESSA framework together, ranking or choosing between a set of options rather than relying on implicit, intuition-based ‘traditional’ decision making ( Belton & Stewart, 2012). By formally structuring and solving decision problems, decision-makers can not only make better decisions (for example by reducing the risks of individual or group biases or simply missing something important) but make decisions better (by laying out explicitly both during the decision-making process and for posterity what criteria have been applied and why, how much weight has been placed upon them, and how scores have been determined). MCDA therefore provides a useful basis for reproducible and auditable decision management, and for facilitating the involvement of a wide range of stakeholders in decision-making, systematically taking account of their preferences, and communicating and justifying the final recommendation taken back to them.

MCDA methods generally involve numerical consideration of:

- **Alternatives:** the options to be ranked or selected from. What are they? How many are there?
- **Criteria:** the qualities against which the alternatives are to be assessed. Are the criteria necessary? Are they sufficient? Are they independent?
- **Weights:** expressing the relative importance of the criteria in reaching the decision.

Answering these fundamental questions and *explaining why* they have arrived at the answers provided, is the task of the other key component in the decision-making process; the **people** making (or providing input to) the decision. The *right people* must be involved, and where necessary *supported*, in assembling their knowledge into a solid framework for making the decision prior to assigning scores or preferences to the alternatives. In other words (in our view), *facilitation* of the decision-making process is just as, and arguably more, important than the algorithm used to process the criteria scoring to rank the alternatives. Nevertheless, a model or algorithm must be chosen. 

### Choosing and using a model

Britest has supported facilitated team-based decision-making activities for over a decade using a software tool which offers three decision models for use, all fed by a common decision set-up stage. These are Analytic Hierarchy Process (AHP) ( Saaty, 1980; Saaty, 1987; Saaty, 2008), Elimination and Choice Translating Reality (ELECTRE) ( Roy, 1968), specifically ELECTRE III ( Roy, 1978), and Multi-Attribute Range Evaluations (MARE) ( Hodgett *et al.,* 2014). AHP is based on making successive pair-wise comparisons within a set of multiple alternatives. ELECTRE is an outranking method which uses fuzzy-based pseudo criteria to classify/eliminate alternatives. MARE takes input ranges (lowest, most likely, and maximum values of the decision variable) and presents the output in the form of uncertainty ranges. 

Whilst all these models for decision making (and more) are established in the literature, in practice our experience has been that MARE has proven to be the most popularly applied, probably because it is relatively simple to understand. It is akin to simple Weighted Sum Methods (WSM) but the capacity to take uncertainty in the inputs into account is advantageous, as it allows a decision-maker to visually interpret their decision results with the associated levels of uncertainty in the outputs as part of the picture. It therefore has huge potential for complementarity with a typical stage-gate based approach to project management, taking advantage of incremental improvements in data accuracy for scoring the criteria as part of a stage gated project life cycle. Well-timed and conducted, MCDA can help avoid abortive work on flawed projects and prevent putting all the effort into a single option too early. This potential is however still not sufficiently realised.

Sometimes teams struggle with the perceived complexity of MCDA, or a reluctance to invest time and effort in the process ( Hodgett *et al.,* 2014). In part, this can arise from the intimidating range of options available in the literature to conduct the process. To reiterate, we believe that to a first approximation most models can deliver a better result than gut-based decision making provided they are used within an open, discursive, facilitated process. On other occasions, MCDA may not be used due to simple lack of awareness or a clash with the organisational culture. These sorts of social barriers can be overcome through education, collaboration with specialists in the field, and changes in sectoral norms about how things are expected to be done. The recent European framework for Safe and Sustainable by Design (SSbD) chemicals and materials for example, suggests the use of MCDA to deal with assessment criteria ( Caldeira *et al*., 2022).

### Timely decision making

Those in the manufacturing process industries responsible for decision-making about innovation choices both at project level (choices of materials, technology and equipment) and portfolio (which projects to prioritise) must enhance their decision-making processes to remain competitive. *Early* decision making is desirable, both to avoid sinking cost and effort into unrealisable projects, and to inform development and evidence gathering needs in projects with a future (assisting experimental design and process modelling for example). Early in the development cycle however, hard evidence upon which to base the evaluation of the alternatives can be scarce, and data, when it is available, uncertain. As outlined above, the MARE model has proven itself to be capable of helping decision-makers deal with uncertainty and reach timely, well-thought through and documented decisions, being used within the Britest membership for decisions on everything from equipment choices and process route selection, through to picking the most appropriate business model for an innovation (Private communications from Britest members, 2013 onwards).

Here we present a further development in WSM-MCDA in the presence of uncertainty, Computed Uncertainty Range Evaluations (CURE), that improves upon the level of discrimination offered by MARE without the computational burden of generating heuristic outcome distributions via Monte-Carlo simulation. We compare the information provided by the new approach to that of existing methods using two previously published case studies from the fine chemical and pharmaceuticals industries respectively as points of comparison. We then go on to demonstrate how the CURE model, implemented in a spreadsheet, can be used to support the use of FESSA as an aid to structured thinking about process sustainability. The case study also demonstrates the utility of the approximation to normality at the heart of the CURE method where numerous criteria are involved in the decision model, and the potential importance of sensitivity analysis in the overall decision-making process.

## Methods

### Calculation of decision outcome scores, probabilities, and limits

The inputs to CURE, identically to MARE, are three consensus-based estimates per alternative for each criterion evaluated: the minimum ( *a*), the maximum ( *b*), and the most likely ( *c*) scores. All criteria should be expressed in terms where a maximum is desirable, or if a minimizing criterion is used the corresponding decision variable is inverted before use in calculations. Should there in fact be no uncertainty about a given score, this can be handled readily by inputting identical values for a, b, and c. The scores can be represented by a triangular distribution where a and b have a probability density of zero and c has probability density $\frac{2}{b–a}$ . The scores are then normalised for each criterion relative to the maximum value across all the alternatives, following the max scale normalisation procedure of Chakraborty and Yeh ^g^.


$$\;x_{ij}^{*}=\frac{x_{ij}}{x_{j}^{max}}=\frac{x_{ij}}{b_{j}^{max}},\;x\;=\;a,b,or\;c\;(1)$$

where $x_{ij}^{*}\;$ is the normalised decision variable for the *i ^th^* alternative with respect to the *j ^th^* criterion, *x _ij_* is the decision variable for the *i ^th^* alternative with respect to the *j ^th^* criterion and $x_{j}^{max}$ is the largest decision variable with respect to the *j ^th^* criterion. Since *b* is the maximum value within the input set for any alternative, the largest value of *b* across all *j* alternatives, $b_{j}^{max}$ is equivalent to $x_{j}^{max}$ .

An important and useful consequence of normalisation is that decision makers can mix objective, quantified scores with numerically expressed but qualitative, subjective judgements within the overall decision structure.

The MARE algorithm simply applies the criterion weights *w _j_* to the normalised values and sums $w_{j}a_{j}^{*}$ , $w_{j}b_{j}^{*}$ and $w_{j}c_{j}^{*}$ for the j criteria to produce the minima, maxima and most likely outcome values for each of the i alternatives. Neither the input nor output triangular distributions in MARE need be symmetrical, but may well be skewed, depending upon the values arrived at by the decision-making team. The output uncertainty limits are set at the extreme combinations (all the maxima and all the minima) of the additive normalised decision variables. For independent, additive criteria however, it is unlikely that the outcome will consist of all the criteria values at the same extreme, thus the MARE method *overstates* the uncertainty in the outcome, and the degree of overlap between the distributions if present, will give an over-pessimistic view of the decision-makers’ ability to discriminate between alternatives.

The originator of MARE has published an improved method, Simulated Uncertainty Range Evaluations (SURE) ( Hodgett & Siraj, 2019). SURE utilises simulations based upon triangular distributions to create a plot which visualises the preferences and overlapping uncertainties of decision alternatives. Instead of independently calculating single values for the minimum, most likely and maximum for each alternative, random deviates are generated based upon triangular distributions and the results presented using a kernel density plot. The utility of the SURE approach is demonstrated clearly by the pharmaceutical industry case study presented by Hodgett and Siraj (2019), however whilst with modern computing power SURE offers a simpler solution than (speculative) convolution-based formulas, it is nevertheless based upon a repetitive, computationally demanding simulation approach, which can present a barrier to widespread uptake by potential non-specialist users. CURE represents a further progression based upon the foundations of MARE and SURE. It retains the advantages of simple decision set-up, comprehensible scoring and the ability to explore the uncertainty in the outcomes, but does so with a light computational overhead, allowing easy implementation in commonplace desktop software (Microsoft Excel). This is achieved by using Central Limit Theorem to approximate the additive combination of several independent weighted criterion scores as a Normal or Gaussian distribution.

Central Limit Theorem states that the sampling distribution from a population will tend towards normality regardless of the shape or skewness of the original population, provided that the sample size is sufficiently large. In strict statistical applications a sample size of at least 30 is commonly quoted as a criterion for what is sufficiently large, however in practice averaging even a relatively small number of samples results in a bell-shaped distribution of sample means which is quite close enough to normality for pragmatic working purposes. In the context of *multiple*-criteria decision analysis, provided the number of criteria being summed is more than a handful, a normally distributed output distribution is a reasonable approximation, especially bearing in mind that the input values are frequently far from analytically precise in nature. Provided that neither the input scores nor weightings are such that one additive component dominates the output result, applying Central Limit Theorem should not produce misleading results. 

The set of scaled input triangular distributions for the *i ^th^* of m alternatives and *j ^th^* of *n* criteria has means


$$\;\mu_{ij}^{*}\;=\;\frac{a_{ij}^{{*}^{2}}+b_{ij}^{{*}^{2}}+c_{ij}^{{*}^{2}}}{3}\;(2)$$

and variances


$$\;\sigma_{ij}^{{*}^{2}}\;=\;\frac{a_{ij}^{{*}^{2}}+b_{ij}^{{*}^{2}}+c_{ij}^{{*}^{2}}–a_{ij}^{*}b_{ij}^{*}–a_{ij}^{*}c_{ij}^{*}–b_{ij}^{*}c_{ij}^{*}}{18}\;(3)$$

where σ denotes the standard deviation.

The equivalent parameters for the output normal distributions of outcome scores *A* are then obtained in the usual weighted sums fashion.


$$\;A_{i}\;=\;\sum_{j=1}^{n}\;w_{j}\mu_{ij}^{*}\;fori\;=\;1,2,...m\;(4)$$

$$\;\sigma_{i}^{A^{2}}\;=\;\sum_{j=1}^{n}\;w_{j}\sigma_{ij}^{{*}^{2}}\;fori\;=\;1,2,...m\;(5)$$

From these parameters, probability density and cumulative probability distributions may be calculated using standard properties of the Gaussian distribution. Adopting a convention widely applied in spectral analysis ^h^ we define uncertainty limits (the minimum difference in outcome scores at which adjacent peaks may be resolved from one another) as the full width at half maximum height distance (FWHM), given for a Gaussian distribution ^i^ by


$$\;FWHM\;=\;2\sigma \sqrt{2In2}\;(6)$$

This corresponds to a 5% risk of the alternative of lower mean outcome score in a pair under comparison ultimately delivering a better outcome than the alternative with higher mean.

### Framework for Early-Stage Sustainability Assessment (FESSA)

There are two levels of criteria in the proposed FESSA structure. At the top level, five main criteria cover the requirement for a sustainable manufacturing process. These are *technical feasibility*, the *business model* (including techno-economics), *supply chain feasibility*, *environmental impact*, and *social impact*. Technical and supply chain feasibility are included as prerequisites for a sustainable operation characterised by its economic, environmental, and social impacts. These top-level criteria are helpful for decision analysis, but each is in practice multi-faceted and too broad in coverage to support a sufficiently focused process of consensus scoring by the decision-making team. Each top-level criterion has therefore been split into several contributory sub-criteria, as shown in Table 1. 

**Table 1.** Framework of criteria at two-Levels for Early-Stage Sustainability Assessment (FESSA). The Framework consists of five top-level criteria C _1_…C _5_, each of which has an associated set of contributory sub-criteria C _n1_ to C _nm_.

C _1_ Technical Feasibility	C _2_ Business Model	C _3_ Environmental Impact	C _4_ Supply Chain Feasibility	C _5_ Social impact
C _11_ Innovation flexibility	C _21_ Improves Net Present Value (NPV)	C _31_ Process safety	C _41_ Supply lead times	C _51_ Satisfies a societal desire, need or necessity
C _12_ Enabling technology	C _22_ Extends existing market share	C _32_ Upstream supply chain sustainable	C _42_ Supplier stability	C _52_ Impact on equality
C _13_ Will it work?	C _23_ Opens access to new markets	C _33_ Downstream supply chain sustainable	C _43_ Resource flexibility	C _53_ How many people will be economically supported?
C _14_ Does it scale?	C _24_ Improves time to commercialisation	C _34_ Supply chain for other manufacturing resources is sustainable	C _44_ Customer lead times	C _54_ Contribution to societal knowledge
C _15_ Is the product fit for purpose?	C _25_ Resilient to existing / new competition	C _35_ On-site manufacturing activities sustainable	C _45_ Availability of equipment, maintenance, support materials and services	C _55_ Job creation
C _16_ Of required purity?	C _26_ Product flexibility	C _36_ Fate of assets at end of use	C _46_ Routes for disposal of wastes	C _56_ Upskilling of workforce
C _17_ Adequately specified?	C _27_ Capacity flexibility	C _37_ Circularity		C _57_ Improves working conditions
C _18_ Acceptable / manageable variability?	C _28_ Feedstock flexibility	C _38_ Location flexibility		C _58_ Improves economic ecosystem
	C _29_ Resilient to Regulatory changes	C _39_ Critical raw materials		C _59_ Improves local business opportunities

Each sub-criterion is in turn supported by further scoring guidance notes to assist the facilitator and decision-making team in rating the alternatives. CURE enables computations based upon this two-tier framework to be managed easily. The process of scoring the relevant sub-criteria (not all criteria will be relevant to every decision) for each alternative can be used readily by a facilitator to guide the team through all the aspects of the alternatives they need to consider and thereby build up a rounded view of their relative overall sustainability.

## Results

### Comparison of Methods

We have taken the input parameters for the previously cited examples of the application of MARE towards route selection ( Hodgett *et al.,* 2014) and MARE/SURE for equipment selection ( Hodgett & Siraj, 2019) and computed results using a spreadsheet implementation of the CURE model in Microsoft Excel to compare the outputs. The goal in the first example, was to provide recommendations on selecting the best route to synthesise a chemical from three viable (proprietary) alternatives, based upon five criteria (product yield, levels of toxins, cost, ease of separation, and odour containment). The model inputs are shown in Table 2.

**Table 2.** Inputs to route selection case study (after Hodgett *et al.,* 2014). “Routes” 1 to 3 refer to three alternative routes to synthesise a chemical, details of which were redacted for reasons of confidentiality.

Alternative	Criterion / Weights (w _i_) / Model Inputs														
	Yield			Toxicity			Cost			Separation			Odour		
	0.339			0.077			0.434			0.127			0.023		
	a _ij_	c _ij_	b _ij_	a _ij_	c _ij_	b _ij_	a _ij_	c _ij_	b _ij_	a _ij_	c _ij_	b _ij_	a _ij_	c _ij_	b _ij_
**Route 1**	78	80	81	87	87	87	79	86	95	19	19	19	8	8	8
**Route 2**	68	70	72	74	74	74	8	10	15	90	90	90	89	89	89
**Route 3**	74.5	75	75.5	9	9	9	20	33	36	81	82	84	30	30	30

**Figure 1.**
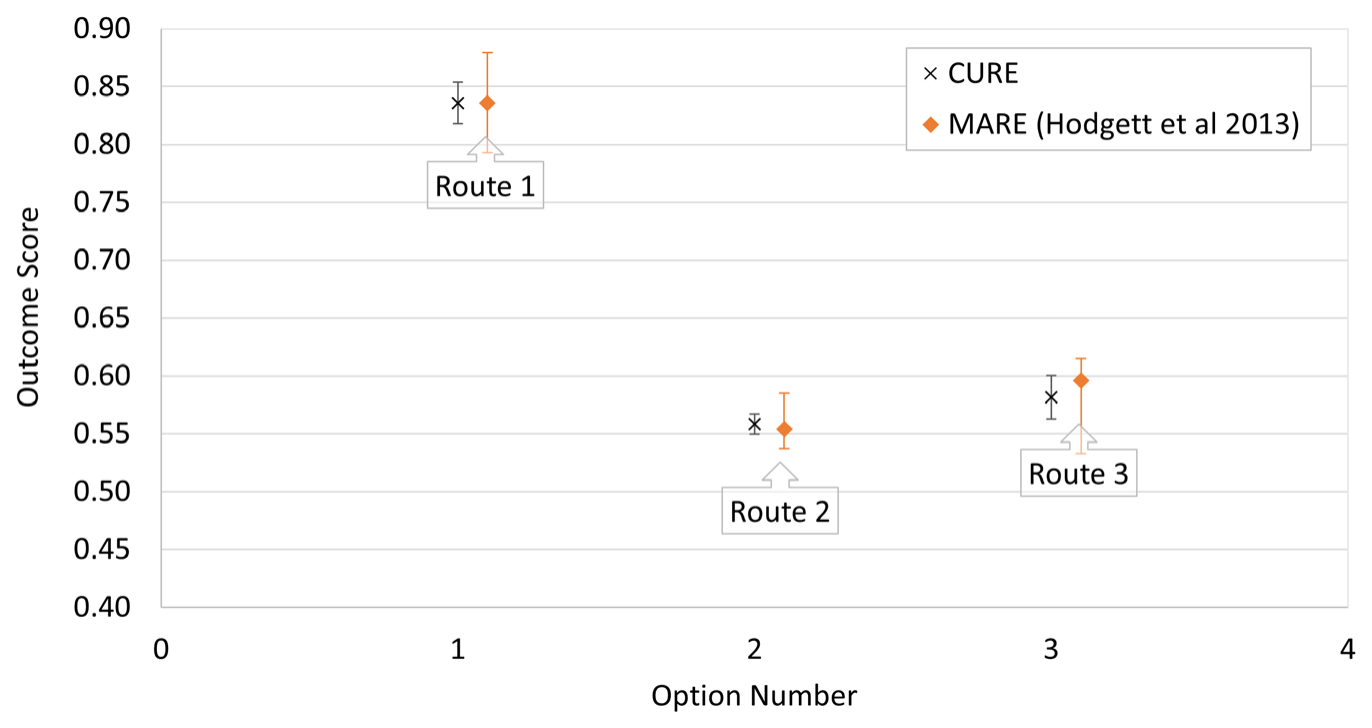
Comparison of CURE and MARE output for Route Selection example of Hodgett *et al.* (2014). The plot compares output summary results from CURE for the three alternatives (black crosses) with their equivalents from the original MARE analysis (orange diamonds). Note that the uncertainty limits for MARE are wider in each case and are unsymmetrical around the most likely value.

Figure 1 shows the output summary results from CURE for the three alternatives alongside their equivalents from the original MARE analysis. The results (indication of the most likely outcomes) are in good agreement but as expected, the uncertainty limits for MARE (given by the weighted sum of the modes of the input triangular distributions) are wider in each case and are unsymmetrical around the most likely value.

For the equipment selection example, the decision challenge arose in the pharmaceuticals industry. This was to select an appropriate degasification technology for a new chemical development process from five alternatives (Packed Column, Membrane, Duty Standby Continuous Stirred Tank Reactor (CSTR)–Vacuum, Duty Standby CSTR with Sparge and Ultrasonic). These were evaluated against five criteria (‘Minimises Hold Up’, ‘Simple to Build’, ‘Technically Possible’, ‘Available Now’, and ‘Low Cost’). Input parameter values are shown in Table 3.

**Table 3.** Inputs to equipment selection case study (after Hodgett & Siraj, 2019). The alternatives correspond to five potential choices of degasification technology for a new chemical development process.

Alternative	Criterion / Weights (w _i_) / Model Inputs														
	Minimises Hold Up			Simple to Build			Technically Possible			Available Now			Low Cost		
	71			26			96			61			50		
	a _ij_	c _ij_	b _ij_	a _ij_	c _ij_	b _ij_	a _ij_	c _ij_	b _ij_	a _ij_	c _ij_	b _ij_	a _ij_	c _ij_	b _ij_
**Packed Column ( *A*_1_)**	49	61	75	58	62	66	-	1	-	87	91	100	69	80	91
**Membrane ( *A*_2_)**	56	88	97	58	70	75	-	1	-	25	76	83	25	80	91
**Duty Standby CSTR –Vacuum ( *A*_3_)**	25	40	48	29	35	51	-	1	-	74	85	97	34	50	59
**Duty Standby CSTR with Sparge ( *A*_4_)**	6	40	48	29	36	52	-	1	-	74	85	97	28	50	59
**Ultrasonic ( *A*_5_)**	45	50	60	4	50	93	-	0	-	0	17	39	3	50	75

**Figure 2.**
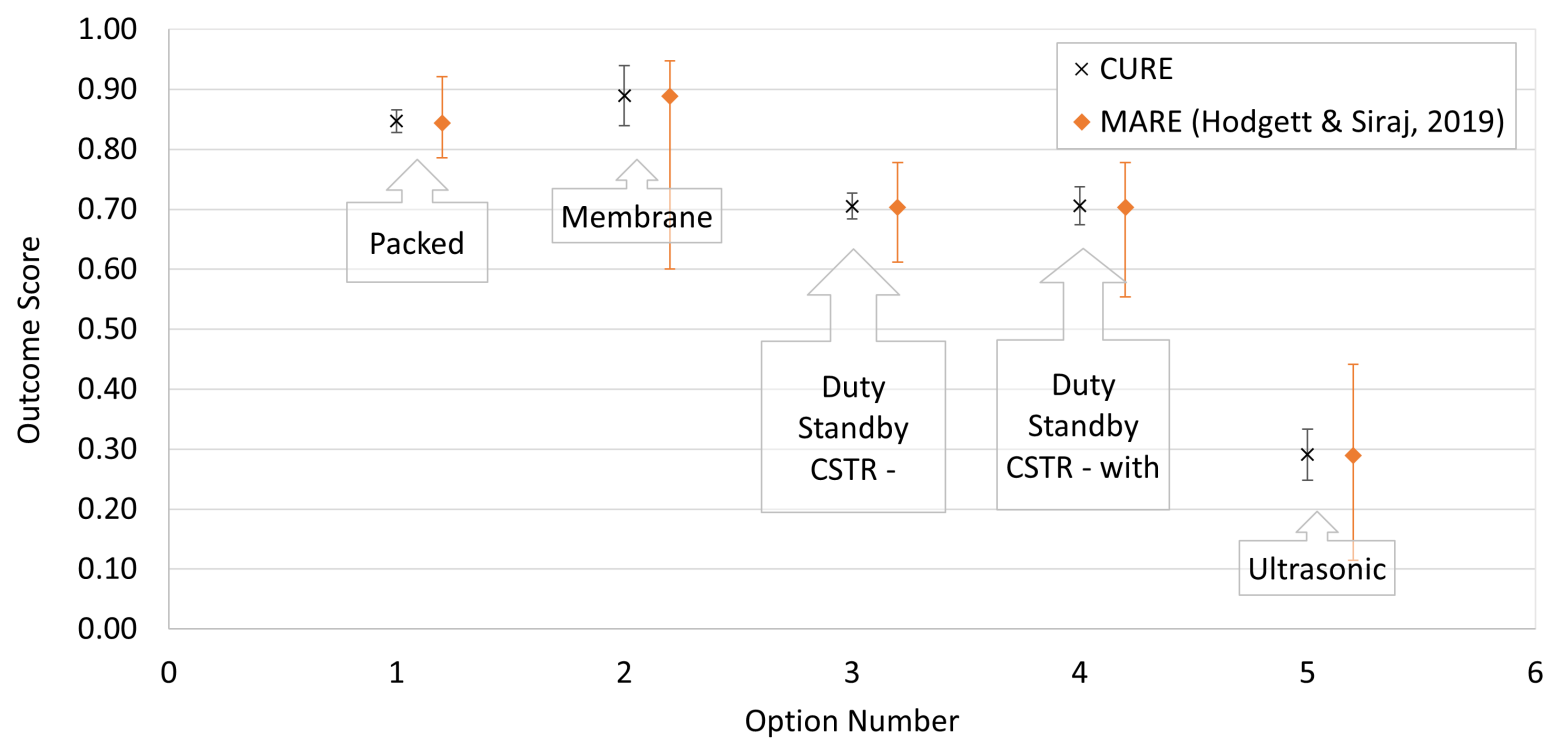
Comparison of CURE and MARE output for Equipment Selection example of Hodgett and Siraj (2019). The plot compares output summary results from CURE for the five alternatives (black crosses) with their equivalents from the original MARE analysis (orange diamonds). Under the MARE model, Alternative 2, Membrane, has especially asymmetric limits. This alternative had input ranges across all four criteria for which ranges were specified that were consistently and widely skewed in the same direction (a≪c<b).

Figure 2 shows the output summary from the CURE evaluation alongside their MARE equivalents. Once again there is good general agreement between the methods, with the corresponding most likely results being close to one another. In most cases the uncertainty limits are considerably narrower for CURE and only slightly skewed for MARE. The most obvious exception to the latter part of this statement is *A*_2_, Membrane which has very asymmetric limits. Inspection of Table 3 shows that that input ranges for this alternative across all four criteria for which ranges were specified are not merely widespread, nor just skewed, but are consistently and widely skewed in the same direction ( *a* ≪ *c* < *b*).

**Figure 3.**
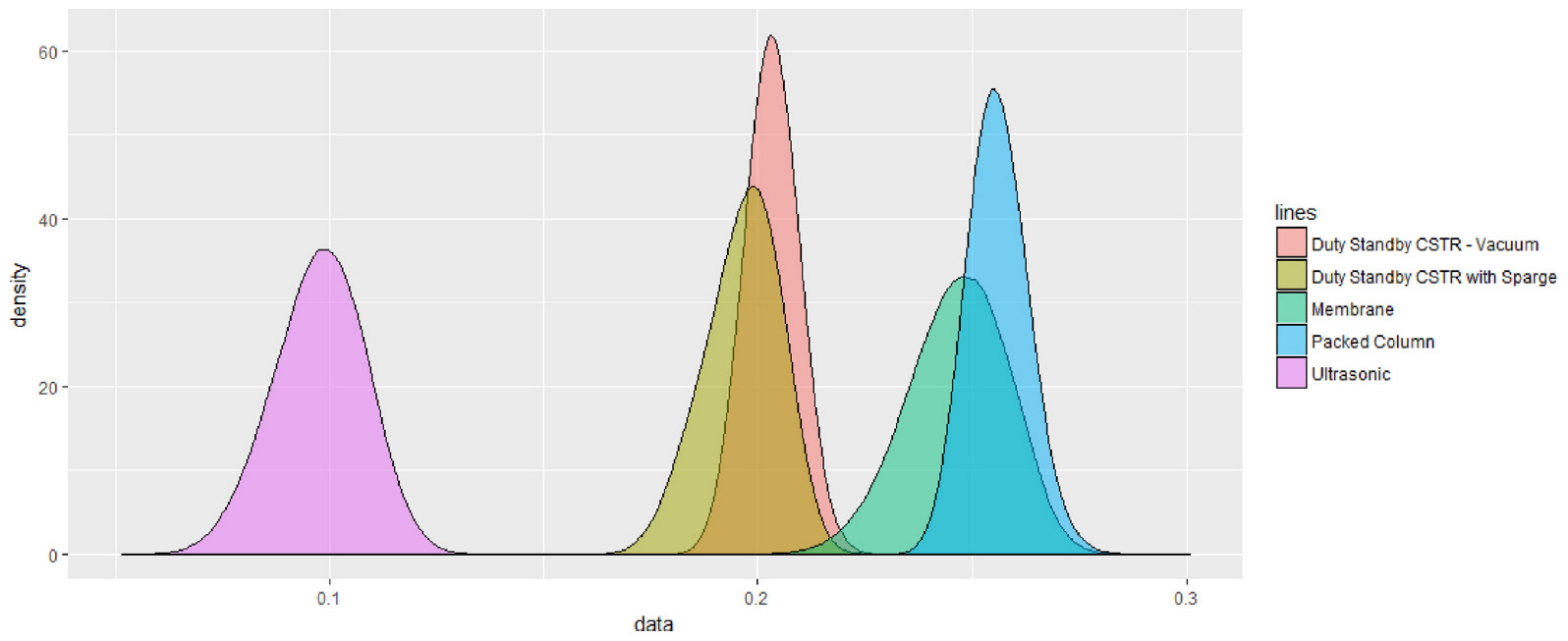
SURE simulated probability densities for equipment alternatives. The curves represent the range of potential outcomes for each of the alternatives under consideration obtained by the SURE simulation method. Reprinted from “SURE: A method for decision-making under uncertainty,” by R.E. Hodgett and S. Siraj, 2019. *Expert Systems with Applications*, 115, 684–694, 2019. ( https://doi.org/10.1016/j.eswa.2018.08.048). Copyright (2019), with permission from Elsevier.

**Figure 4.**
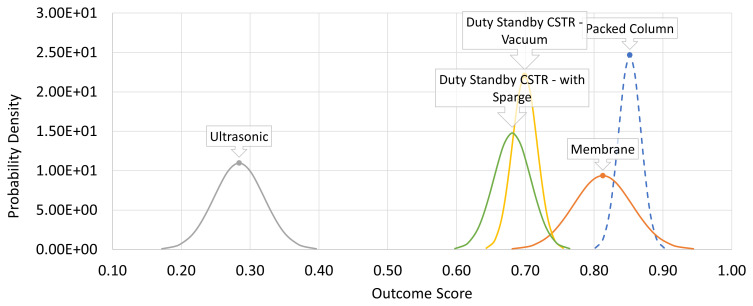
Parameter-derived (CURE) probability density distributions for the equipment alternatives. The curves represent the range of potential outcomes for each of the alternatives under consideration obtained by the CURE approximate calculation method. The plot is qualitatively similar to Figure 3 albeit with arbitrarily different scaling of the abscissa. In either case, the higher the output score value, the more positive is the prognosis.

In Hodgett and Siraj (2019), the authors’ analysis continued using the SURE method to generate a simulation-based kernel density plot, Figure 3. The corresponding normal probability density plot generated by the CURE spreadsheet is shown in Figure 4.

The plots show qualitative similarity albeit the scaling of the abscissa is arbitrarily different. The main refinement in information provided by the non-approximation based SURE method is to highlight skewness in the output distribution, as is seen with the membrane option in this case. Nevertheless, the plots also indicate that in the central, more probable region of the distributions, the CURE and SURE curves are reasonably alike. The CURE output successfully conveys a good indication of the degree to which uncertainty is more or less of a factor in the outcome generated.

### Case study: Process technology selection for a next-generation superabsorbent

We now demonstrate the retrospective application of the FESSA framework and the CURE method to a real-world historic process technology selection decision faced by a manufacturer of speciality polymers.

***The decision context.*** Cross-linked sodium polyacrylate is the primary active absorbent material in personal hygienic devices such as disposal nappies (‘diapers’), adult incontinence garments, and feminine hygiene products. Typically used in solid granular form, superabsorbent polymer (SAP) may absorb of the order of 20 to 50 times its own mass of aqueous electrolytes such as saline, urine or blood. The quality-critical swelling capacity versus extractable (uncrosslinked, soluble polymer) content relationship is defined by the quality of the molecular network assembled during the polymerisation reaction. The process chemistry involved in producing SAP at high tonnage commercial scale involves choices and trade-offs between process space-time, energy input per unit output and the resultant product properties obtained. 

The case study presented here reflects a historic (mid-1990’s) decision faced by a commercial manufacturer of SAP who, having established a market position with a cost-competitive first-generation product, wished to expand their market share by gaining access to the market-leading users of SAP in the global disposable hygienics industry. These customers expected a fundamentally higher level of technical performance than was achievable using the company’s first-generation product platform technology. The incumbent approach ^j^ took advantage of inherent energy-efficiency in the process concept to make a cost-effective product offering, however there was no window within the existing process limits which could deliver the package of performance quality demanded by the prospective premium customers. *A choice had to be made* about which potential approach or approaches should be prioritised to meet this need.

The decision alternatives considered here reflect the contemporary R&D portfolio of the company. Whilst not all the alternatives were direct candidates for meeting the immediate business need just described they are helpful in illustrating how FESSA and CURE can elucidate how alternatives differ not just in the overall outcome score, but also how well or otherwise they address different criteria within the overall assessment. The alternatives were:

***A*_1_: High concentration thermal polymerisation**. The incumbent process, in which the use of a high monomer concentration meant that the exothermic heat of reaction could be used to drive water away from the formative polyacrylate resin so that it could be reduced to a powder by pulverisation without an intermediate forced drying step. The polymerisation reaction timescale was less than a minute, but further time was provided to allow the bulk polymer to cool and become sufficiently brittle prior to size reduction.

***A*_2_: Increased degree of neutralisation**. A formulary variant on the existing process whereby the degree of neutralisation of the acrylic acid monomer is increased towards the known quality-optimal value (~75%) with some modest trade-off in process space-time requirements.

***A*_3_: Thermal gel polymerisation**. At significantly lower monomer concentrations polymerisation proceeds over the course of about an hour to produce a hot gel. The more moderate reaction conditions result in a closer to ideal polymer network. Further energy must however be expended to remove water prior to milling by extruding and then drying the gel by forced warm air circulation.

***A*_4_: Inverse suspension polymerisation**. Acrylic monomers in aqueous solution can be suspended in a suitable hydrophobic organic external phase and polymerised into spherical beads of a size suitable for direct use as superabsorbents once water has been removed from them by forced-air drying. Although mechanical size reduction requirements are minimal, there are yield losses due to over- and under-sized dry bead production.

***A*_5_: Non-thermal gel polymerisation**. A well-moderated gel reaction yielding quality benefits like those of A _3_ could be achieved with a substantially shorter reaction time using an alternative reaction initiation package which did not require thermal activation.

***A*_6_: Starch based SAP**. Lab scale samples of a superabsorbent powder derived entirely from a plant-based source (potato starch) had been provided by a university for evaluation. Only limited information on the associated process / chemistry was known or could be inferred.

***A*_7_: Microwave based polymerisation**. A method whereby a monomer formulation like that of the incumbent process could be polymerised within a microwave field to produce SAP directly and rapidly in the form of light, fine flakes had been shown to be feasible in a small-scale batchwise, benchtop demonstration.

***Allocation of Weights.*** The weights applied to the five top level criteria of the decision support model, Table 4, were set to reflect the contemporary business priorities. The accompanying comments on the rationale behind the weights given were recorded prior to considering the alternatives in detail against the associated sub-criteria.

**Table 4.** First-level criteria and weights for SAP case study. The indicated weights were set to reflect the contemporary business priorities. The accompanying comments on the rationale behind the weights given were recorded prior to considering the alternatives in detail against the associated sub-criteria.

Criterion	Weight	Rationale
***C*_1_ Technical** ** Feasibility**	30%	The primary driver for innovation was to make a step change in product quality to access premium customers, which would substantially increase market share in the existing market. Clearly this also needed to be via a commercially viable process. Some interesting alternatives are also considered which do not necessarily fit this target, so they are assessed on their own merits against relevant modified targets and specifications.
***C*_2_ Business ** **Model**	25%	Important, though perhaps in many cases (except the longer-term R&D options) liable to be less of a differentiator since the main options would operate through essentially the same business model.
***C*_3_ Environmental ** **Impact**	20%	Primarily important in terms of process safety and local environmental impacts at the time, this would nowadays arguably weigh rather higher.
***C*_4_ Supply Chain** ** Feasibility**	15%	Mostly related to raw materials specifications for quality/operability resulting in any limitations on supply options.
***C*_5_ Social Impact**	10%	Primarily related to benefits of products to society, workforce safety, operating conditions, and some aspects of benefit for local economies.

Sub-criteria weights determined in a similar fashion are shown in Table 5. The corresponding rationales for each sub-criterion may be found in the companion data set to this paper ( Henderson & Peeling, 2024). Only one sub-criterion *C*_38_ (Use of Critical Materials) was deemed irrelevant and thus allocated a zero weighting.

**Table 5.** Sub-criterion weights for SAP case study. The corresponding rationales for each sub-criterion may be found in the companion data set to this paper ( Henderson & Peeling, 2024). Sub-criterion C _38_ (Use of Critical Materials) was deemed irrelevant and thus allocated a zero weighting.

Criterion	Sub-criterion / Weight								
***C*_1_**	*C*_11_	*C*_12_	*C*_13_	*C*_14_	*C*_15_	*C*_16_	*C*_17_	*C*_18_	
	8%	4%	16%	16%	16%	16%	8%	16%	
***C*_2_**	*C*_21_	*C*_22_	*C*_23_	*C*_24_	*C*_25_	*C*_26_	*C*_27_	*C*_28_	*C*_29_
	10%	19%	10%	10%	19%	10%	10%	10%	5%
***C*_3_**	*C*_31_	*C*_32_	*C*_33_	*C*_34_	*C*_35_	*C*_36_	*C*_37_	*C*_38_	*C*_38_
	25%	13%	13%	13%	19%	3%	3%	13%	0%
***C*_4_**	*C*_41_	*C*_42_	*C*_43_	*C*_44_	*C*_45_	*C*_46_			
	13%	25%	25%	13%	13%	13%			
***C*_5_**	*C*_51_	*C*_52_	*C*_53_	*C*_54_	*C*_55_	*C*_56_	*C*_57_	*C*_58_	*C*_59_
	28%	7%	14%	7%	7%	7%	14%	7%	10%

All criteria were assessed for each of the alternatives by use of qualitative scores on a scale of 0 to 1 where 1 represented an ideal outcome. In each case the input uncertainty was stated in terms of the parameters of a triangular distribution, and the reasons for the assessment recorded. Full details are contained in the companion data set ( Henderson & Peeling, 2024).

**Figure 5.**
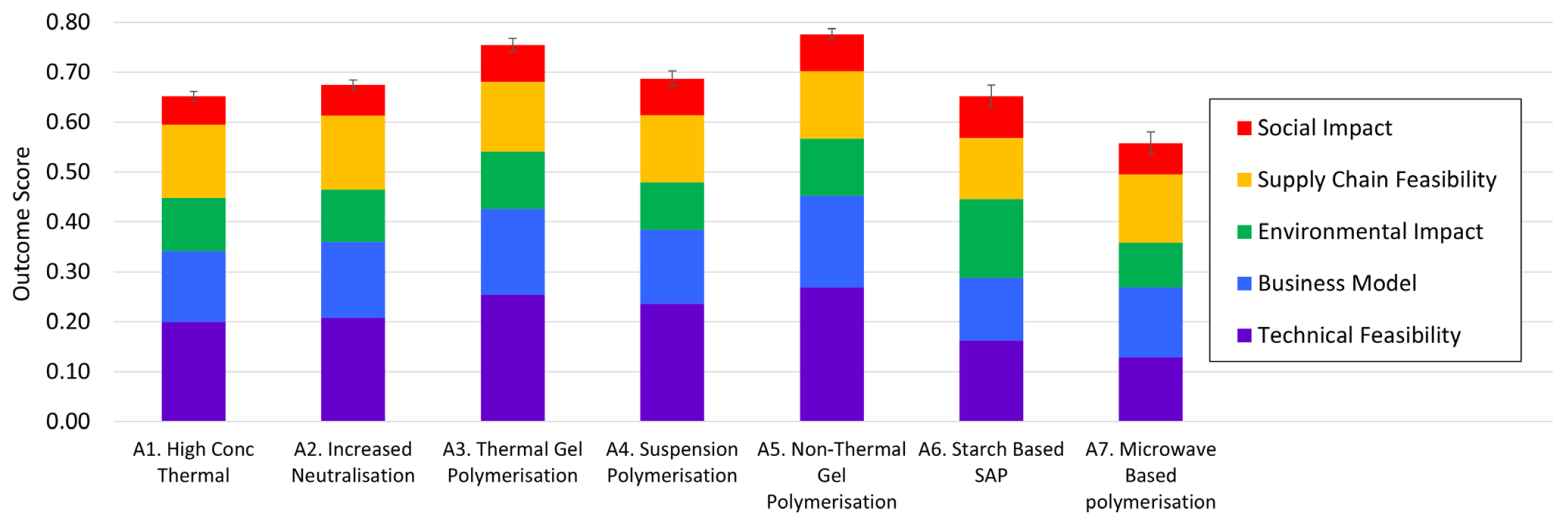
Contributions to SAP case study decision support output scores. Error bars indicate the Full-Width Half-Maximum (FWHM) distance, corresponding to a 5% risk in a pairwise comparison that the alternative of lower score would ultimately delivering a better outcome than the one with higher score, based on the input uncertainty ranges.

***Decision Scores.*** The intermediate criterion scores *C*_1…5_, final overall outcome scores *A*_1…7_, and uncertainties shown in Table 6 and Figure 5 were obtained using Equation (1) to Equation (6).

**Table 6.** Summary of decision support output scores. The intermediate criterion scores *C*_1…5_, final overall outcome scores *A*_1…7_, and uncertainties were obtained using Equation (1) to Equation (6).

Alternative	*C*_1_	*C*_2_	*C*_3_	*C*_4_	*C*_5_	Output Score	Combined SD (σ)	Uncertainty $\left(\pm \sigma \sqrt{2\ln2}\right)$
***A*_1_**	0.199	0.143	0.106	0.147	0.056	0.651	0.0083	0.010
***A*_2_**	0.208	0.152	0.106	0.147	0.061	0.674	0.0088	0.010
***A*_3_**	0.255	0.171	0.115	0.140	0.073	0.754	0.0110	0.013
***A*_4_**	0.237	0.147	0.095	0.134	0.074	0.687	0.0128	0.015
***A*_5_**	0.269	0.184	0.114	0.135	0.074	0.776	0.0095	0.011
***A*_6_**	0.162	0.125	0.159	0.123	0.083	0.652	0.0184	0.022
***A*_7_**	0.129	0.140	0.089	0.137	0.063	0.558	0.0185	0.022

From Figure 5 it is easily seen that there are two favoured alternatives, *A*_3_ and *A*_5_, the thermal and non-thermal variations respectively of a gel polymerisation process. Although the uncertainty limits for the two alternatives overlap with one other, the error bars do not overlap with those of any of the other alternatives. *A*_5_ combines the highest overall mean (most likely) outcome with one of the lowest uncertainties in that outcome. This was in fact the alternative adopted by the company at the time.

**Figure 6.**
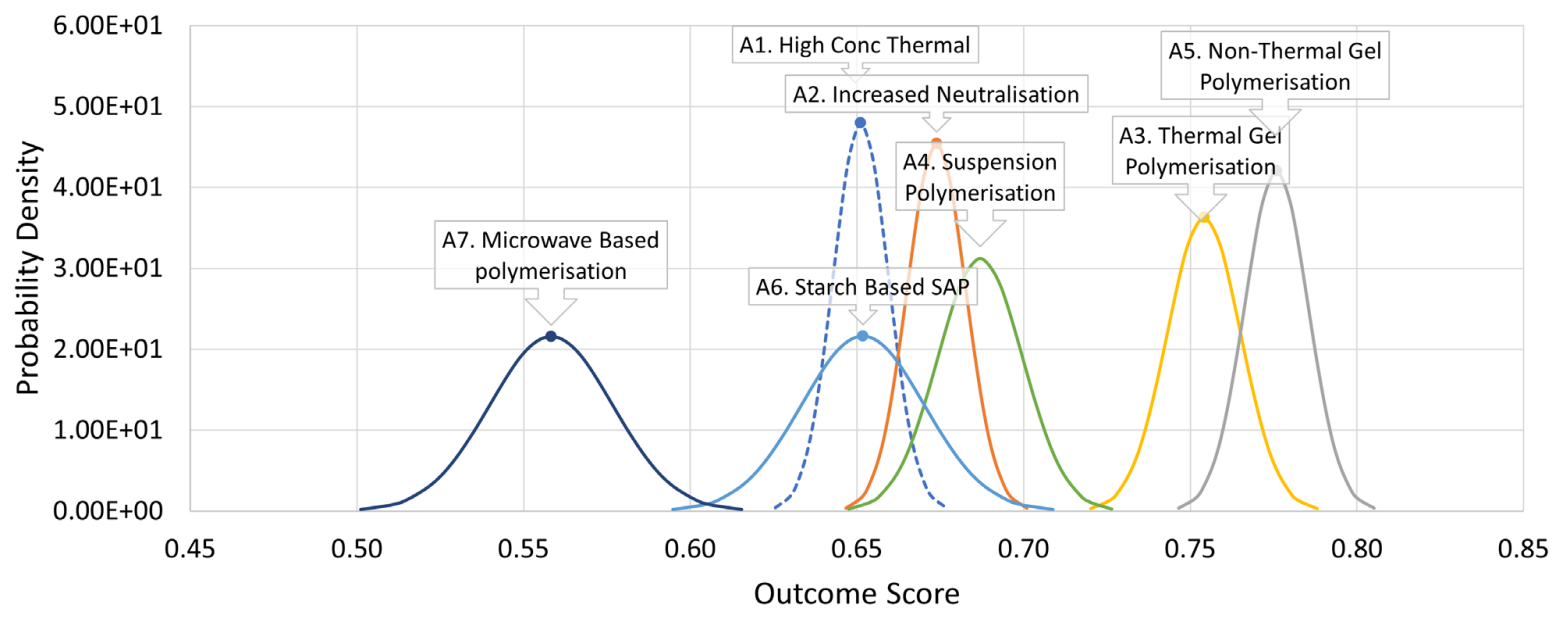
Probability density plots for SAP case study decision outcomes. The incumbent option is shown with a dashed line to facilitate relative comparison with the alternatives.

Figure 6 summarises the probability density distributions of the estimated outcomes based on the weighted combination of all the input parameters. Again, the pre-eminence of *A*_3_ and (especially) *A*_5_ is quite clear.

From Figure 6 it is evident that whilst *A*_5_ is the alternative most likely to lead to the best outcome, there remains a small but non-negligible possibility that whatever outcome were ultimately to be attained by pursuing *A*_5_ that it would have been exceeded by *A*_3_ had that been chosen instead. This may be thought of as the “downside” risk of choosing *A*_5_ over *A*_3_. Of course, there is a more than counterbalancing “upside” *opportunity*. Clearly it is *more* likely that *A*_5_ will deliver a *better* outcome than *A*_3_. These concepts may readily be quantified for normally distributed outcome distributions.

For any actual outcome value *x* obtained for the alternative proposed to be adopted, *A* (in this example *A*_5_), the downside risk and upside opportunity associated with another option not taken, *O* (in this case *A*_3_), are given respectively by


$$Downside\;=\;p_{A}\left(x\right).p_{o}\left(>x\right),\;\mathrm{and}\;Upside\;=\text{​}\;p_{A}\left(x\right).p_{o}\left(\leq x\right)\;(7)$$

where *p* represents a probability derived from the Normal distribution for the indicated event.

**Figure 7.**
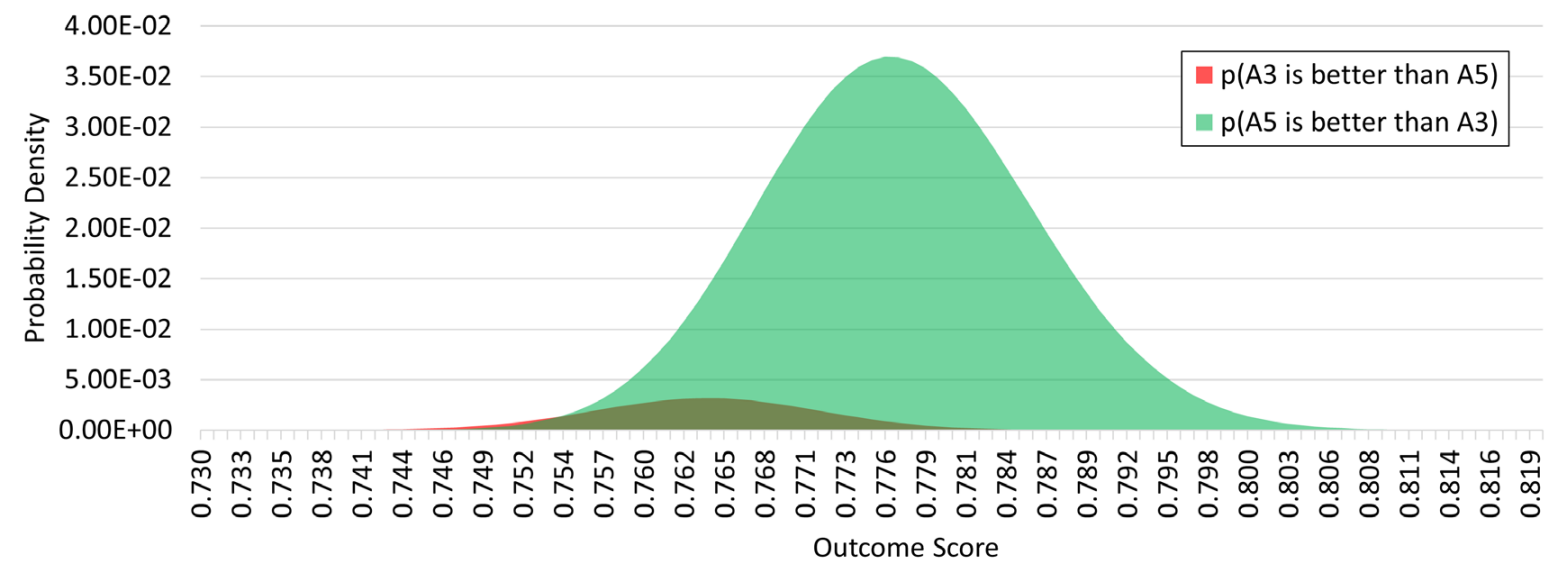
Probability density plot for decision outcomes considering only alternatives A3 and A5. The area under the red curve represents the risk that the actual outcome attained by pursuing A _5_ would have been exceeded by A _3_ had that been chosen instead, in other words the “downside” risk of choosing A _5_ over A _3_. The area under the green curve represents the corresponding “upside” probability that A _5_ is in fact the better choice.

The probability densities of these two quantities for the case of *A*_5_ vs *A*_3_ are shown in Figure 7. Summing over all values of x gives the overall “balance of probabilities” which for this example gives an upside opportunity of 94.5% versus a downside risk of 5.5%.

**Figure 8.**
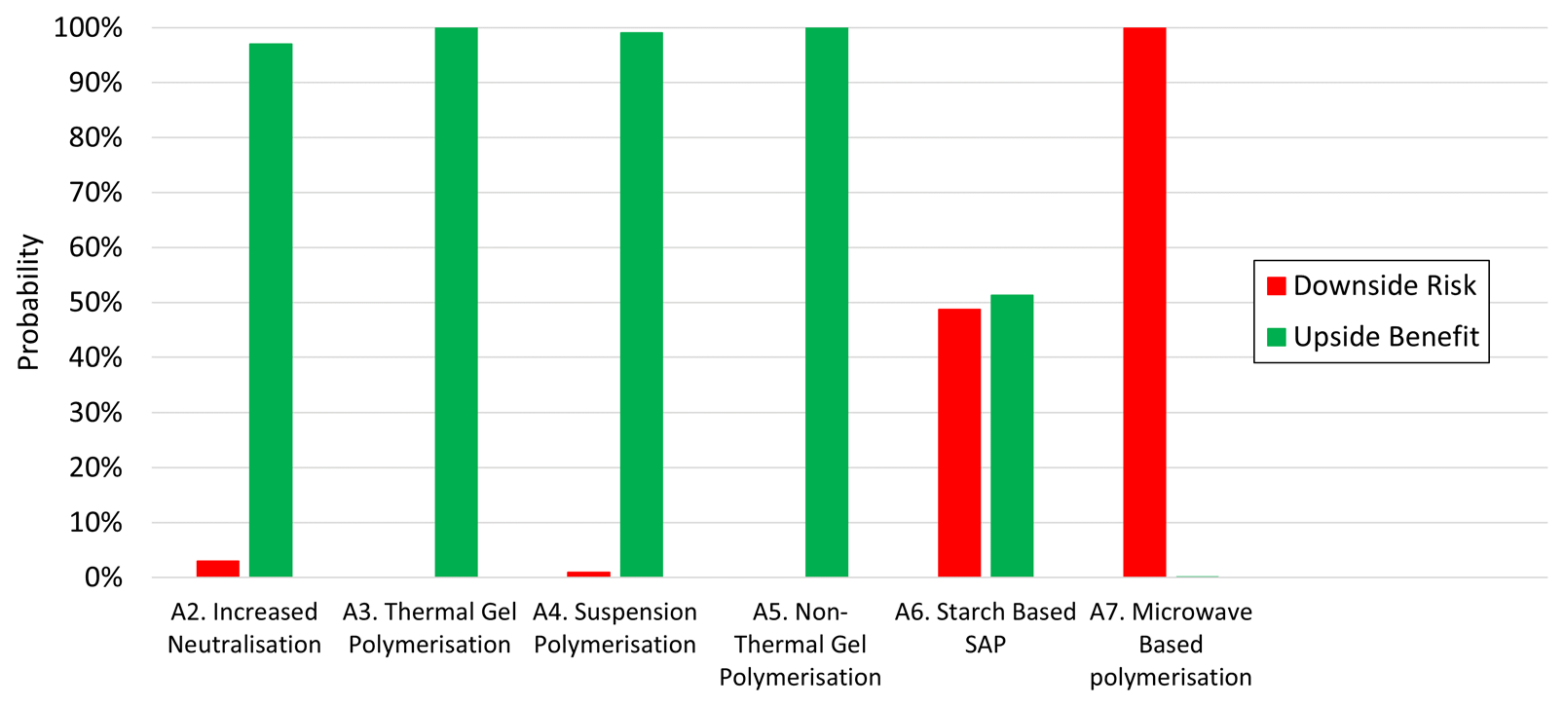
Balance of probabilities summary. Each pair of bars represents the balance of probabilities between the incumbent A _1_ and one of the alternatives. A large green bar indicates in favour of change, a large red bar towards the *status quo*.

Returning to the overall premise of the case study, a useful comparison may be made between the balance of probabilities for each of the alternatives *A*_2_ to *A*_7_ reckoned against staying with the status quo, *A*_1_. The down- and upside probabilities for each of the alternatives are conveniently summarised in a bar chart, Figure 8. In each case the red bar indicates the probability that staying with the incumbent *A*_1_ would result in a better outcome than adopting the alternative, whilst the green bar represents the corresponding upside probability of realising a better outcome by going with the alternative.

## Discussion

### Normality: a satisfactory approximation?

The comparison provided by the earlier equipment selection example, which featured five criteria (of which only four were range-based) illustrates both the usefulness of CURE as a “good-enough for guidance” tool in decision-support but also its, generally tolerable, limitation with small numbers of criteria. This limitation may be expected to diminish in situations where there are more criteria, where the outcome distribution would be expected to tend closely towards normality. This can readily be demonstrated by simulation based upon the FESSA framework-based SAP case study. To test whether it is appropriate to approximate normality in the outcomes we have generated simulated decision outcomes (based on a sample size of 5000) as follows.

Starting from a random variate *U* drawn from the uniform distribution in the interval (0, 1), a random variate *X* for the triangular distribution in the same interval may be generated by


$$X\;=\;\left\{ \begin{array}{l} a+\sqrt{U(b–a)(c–a)}\;for\;0<\;U\;<\;F(c)\\ b–\sqrt{\left(1–U)(b–a)(b–c\right)}\;for\;F(c)\leq U<1\;(8) \end{array}\right.$$

where *F*( *c*) = ( *c*– *a*)/( *b*– *a*) and *a*, *b* and *c* are the three characteristic values of the triangular distribution as previously defined.

Using the same criterion weights and input values for specimen alternatives drawn from the case study, weighted sums of the relevant triangular random deviates can be obtained for the five intermediate first level criteria and summed in turn according to the weights applied to provide simulated outcome distributions. We have analysed how closely the resulting distributions approximate to normality using statistical significance tests for skewness (lack of symmetry) and kurtosis (deviation from the characteristic normal distribution shape with respect to the weight of the tails relative to the peak). Specimen results for one alternative are shown in Table 7, including significance test results for examples of symmetric, and positively / negatively skewed input triangular variates for comparison. The additive statistical error in the complete set of component triangular deviates produces five intermediate top-level criterion score distributions which display modest deviations from normality. Two criteria, *C*_1_ and *C*_4_, display statistically significant negative skewness, and all bar *C*_2_ are significantly platykurtic (displaying more weight in the tails than the Normal distribution). When these in turn are combined into the outcome variate, the distribution displays neither significant skewness nor kurtosis.

**Table 7.** Significance test results for skewness and kurtosis in simulated score distributions. Results are median values for D’Agostino-Pearson tests for Alternative A _5_ derived from 8 independent simulations for each score. p > 0.05 or confidence interval limits straddling zero indicate a non-significant result, i.e., close agreement with normality.

Variate	Skewness			Kurtosis		
	p	Lower Limit	Upper limit	p	Lower Limit	Upper Limit
***C*_17_**	0.294	-0.067	0.069	**0.000**	-0.719	-0.448
***C*_21_**	**0.000**	-0.494	-0.358	**0.000**	-0.727	-0.456
***C*_45_**	**0.000**	0.501	0.637	**0.000**	-0.743	-0.471
***C*_1_**	**0.003**	-0.162	-0.027	**0.024**	-0.273	-0.001
***C*_2_**	0.062	-0.121	0.014	0.056	-0.247	0.025
***C*_3_**	0.110	-0.110	0.025	**0.012**	-0.293	-0.021
***C*_4_**	**0.000**	-0.312	-0.176	**0.032**	-0.264	0.007
***C*_5_**	0.342	-0.067	0.069	**0.019**	-0.279	-0.008
**A _5_** **(Outcome)**	0.072	-0.119	0.017	0.310	-0.149	0.123

### Sensitivity analysis

The decision arrived at, that *A*_5_ is substantially the best alternative with *A*_3_ as a good back-up, is clear enough given (i) what was known contemporaneously about the alternatives and (ii) the relative importance of the criteria expressed through their weights. As mentioned above, this indeed was the strategy that was successfully adopted by the manufacturer at the time. It is however fair to observe that, in business and society at large, awareness and prioritisation of matters such as the environmental impacts and social equity of manufacturing have risen substantially in the intervening years. Thus, it is possible that, viewed through a contemporary lens, a greater emphasis on these aspects of the decision model might lead to a different indication of “the best” alternative(s). 

Addressing the question of the robustness of the decision (would a different conclusion have been reached if different weights had been applied?) is the objective of sensitivity analysis. Without further data or evidence gathering, the consensus *scores* allocated to the alternatives against sub-criteria should not be changed. In principle, one could go back and investigate the impact of varying the weights applied at the sub-criterion level upon the decision outcome. In practice, the difference made to the output score caused by even moderate changes in one amongst many additive contributions would be modest, and to consider them collectively would be a cumbersome exercise. Instead, we limit our attention to the impact of altering the weights applied to the five top-level criteria.

Sensitivity in the outcome to criterion weights is only possible if there are proportionately different contributions from the criteria to the overall result across the different alternatives. Figure 5 is helpful in gauging this. The proportional contributions of the stacked bars to the overall bar height (output score) vary across the alternatives. Sensitivity analysis is therefore worthwhile.

**Figure 9.**
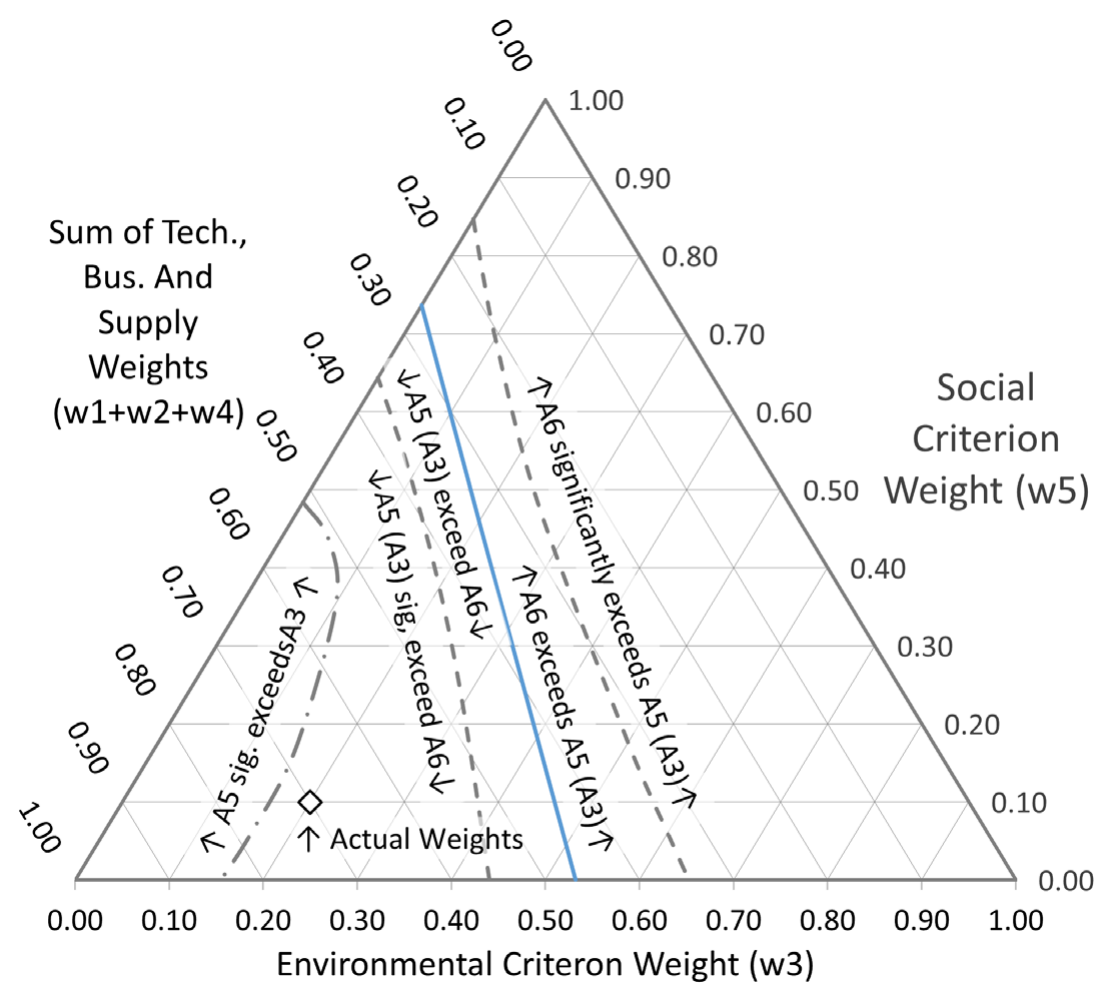
Sensitivity plot. The primary and secondary axes represent *w*_3_ and *w*_5_, the weights allocated to the Environmental and Social Impact criteria respectively. w _1_, w _2_ and w _4_ are set at their original relative proportions (30:25:15), and together are represented by the distance along the tertiary axis, which is the difference between the sum of w _3_ and w _5_ and 1. The blue line represents the boundary at which A _6_ becomes preferred over A _5_ (or A _3_). The dashed grey lines indicate the positions where this change becomes statistically significant in either direction. The line of alternating long and short dashes identifies where A _5_ becomes resolved from A _3_. The point marked with a white diamond shows the position of the actual weights applied.

The high outcome scores for *A*_5_ and *A*_3_ arise primarily because they were allocated very high scores on the Technical ( *C*_1_) and Business ( *C*_2_) criteria. They generally score well for the other three criteria, but neither is the best in any of them. *A*_6_ (the starch-based SAP) by contrast is substantially the highest scoring on the Environmental criterion ( *C*_3_) and, by a narrower margin, on Social Impact ( *C*_5_). The highest scores for Supply Chain aspects ( *C*_4_) were obtained for the incumbent, and its iterative variant *A*_2_, primarily because these options involve known technology and known customers. By varying the weights *w _j_*, it is possible to identify boundary conditions where the first ranked alternative switches from one candidate to another and thus construct a diagram analogous to a chemical phase diagram, wherein the lines indicate the boundaries between regions of first preference according to the criterion weights applied. Here we regard *A*_3_ and *A*_5_ as an equivalent tied-best pair and compare the nominally higher scoring of them against the next best option *A*_6_. Such a plot for the SAP system is shown in Figure 9.

The primary and secondary axes represent *w*_3_ and *w*_5_, the weights allocated to the Environmental and Social Impact criteria respectively. The total of the five weights must sum to one, thus the sum of the remaining three criteria is obtained by subtracting the sum of *w*_3_ and *w*_5_ from 1. This is represented by the tertiary axis. This quantity is divided amongst *w*_1_, *w*_2_ and *w*_4_ in the originally applied proportions (30:25:15). Thus, the point marked with a white diamond at co-ordinates (0.2, 0.1, 0.7) shows the position of the actual weights applied. The blue line on Figure 9 representing the boundary at which *A*_6_ becomes preferred over *A*_5_ (or *A*_3_) lies a long way from the applied weightings. There would have to be a good deal more emphasis placed upon both the environmental and social aspects of the innovation for a different decision to be indicated. The decision made is, in other words, essentially robust. We can however usefully extend the analysis.

One of the main attractions of MARE, SURE and CURE is their capacity to take uncertainty in the scoring into account. Therefore, in addition to the simple *A*_5_ = *A*_6 _boundary just discussed, we have also identified further boundaries, where the lower limit of uncertainty for the leading alternative equals the upper limit of uncertainty of the next best alternative. These are shown by the dotted curved bands either side of the blue line and correspond to conditions where the downside risk associated with the leading alternative is 5%. In this case, the location of the significance band for *A*_6_ is even further towards the top and right of the diagram, emphasising the robustness of the basic decision. On the other hand, the line defining the boundary of the region where *A*_5_ is significantly better than *A*_6_ runs rather closer to the actual weights applied. This serves as a reminder that *A*_6_ is not so far from entering consideration as might appear to be the case at first glance. Finally, a further boundary (the line of alternating long and short dashes in Figure 9) may be identified where *A*_5_ becomes resolved from *A*_3_. The boundary of this region passes fairly closes to the point of the actual weights applied, so if (for example) the Environmental Impact criterion had been weighted at 0.1 instead of 0.2, the final analysis would have declared *A*_5_ unambiguously better than *A*_3_. 

**Figure 10.**
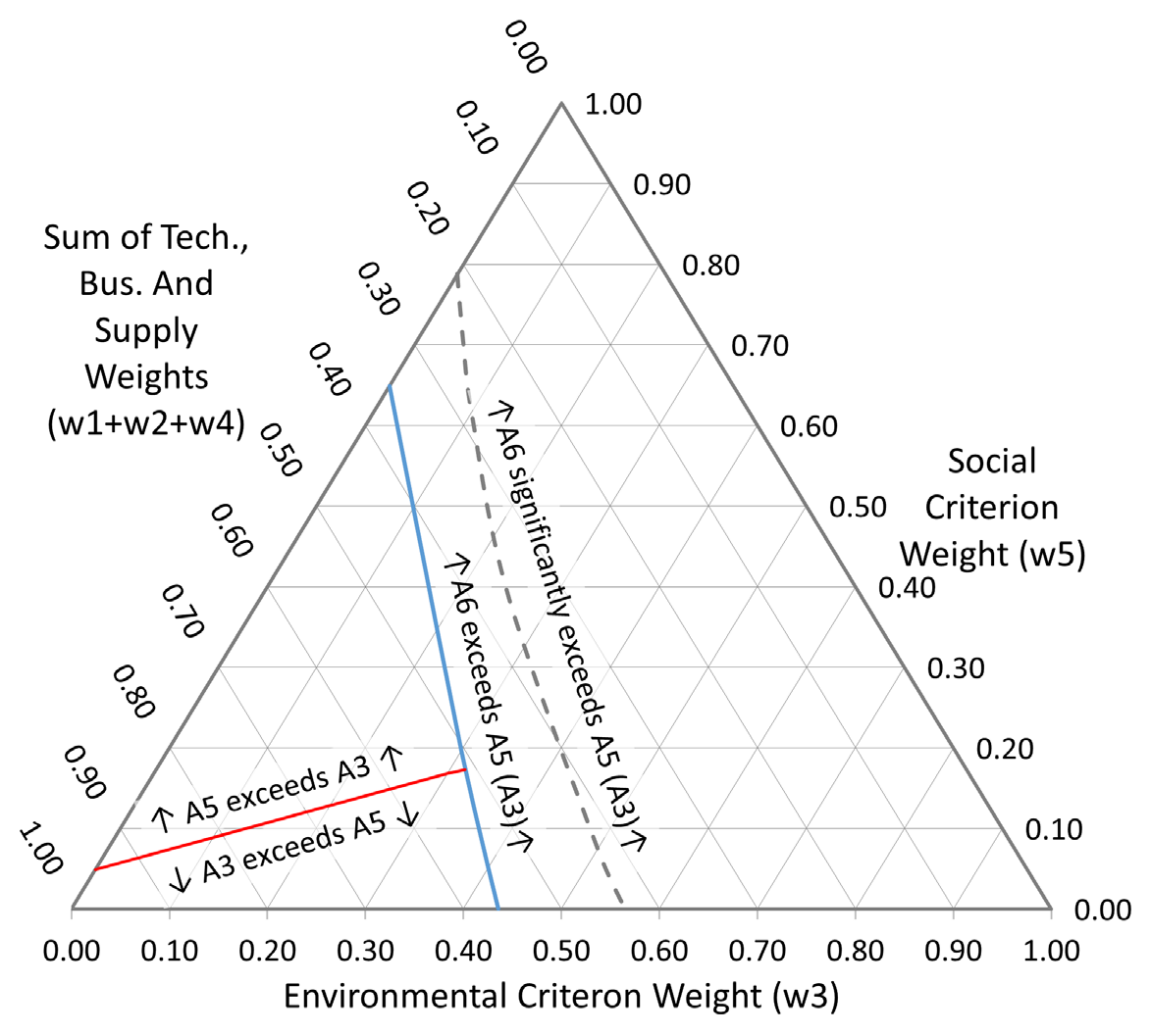
Sensitivity plot with w1:w2:w4 = 1:1:3. The blue line represents the boundary at which A _6_ becomes preferred over A _5_ (or A _3_). The dashed grey line shows where the change becomes statistically significantly in favour of A _6_. The red line indicates where the order of 1 ^st^ preference changes between A _5_ and A _3_.

The sensitivity of the decision outcome changes with further shifts in the relative weights applied to the criteria. Placing more importance on the Supply Chain Feasibility criterion (increasing *w*_4 _relative to *w*_1_ and *w*_2_) tends to shift the previously identified boundary lines towards the lower left corner of the diagram, and a third region emerges where the order of preference of *A*_3_ and *A*_5_ is inverted (though not significantly so), see Figure 10.

**Figure 11.**
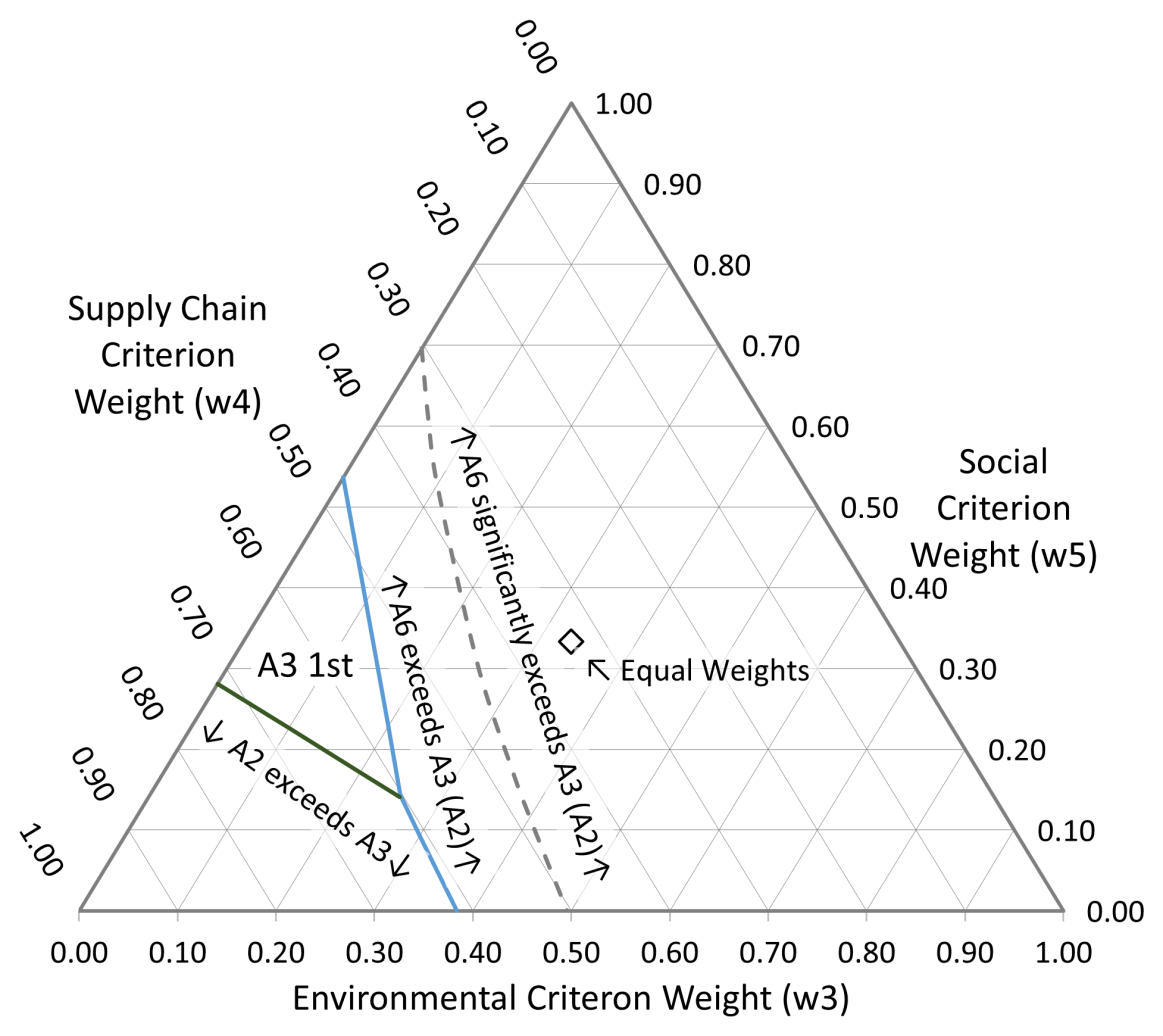
Sensitivity plot with w _1_ and w _2_ = 0. At the point labelled ’Equal Weights’ *w*_3_ = *w*_4_ = *w*_5_ = 1/3. The blue and green solid lines indicate regions of 1 ^st^ preference change between A _6_ and A _3_/A _2_ and between A _3_ and A _2_ respectively. The dashed grey line shows where the change becomes statistically significantly in favour of A _6_. Note that at the point of equal weighting for the three remaining criteria, shown by the white diamond, A _6_ is the clear first choice.

In the extreme case where the Technical and Business criteria are excluded by setting *w*_1_ and *w*_2_ to zero ( Figure 11), *A*_2_ enters the picture as a first ranked alternative, in the corner of the diagram where the Supply Chain criterion is dominant. The primary attraction of this alternative was that it offered a rapid path to implementation, albeit with only commensurately modest quality gains. This option was in fact used by the business, essentially to extend the lifetime of the incumbent technology platform whilst longer term solutions were pursued.

In Figure 11 we may also note that, at the point of equal weighting for the three remaining criteria, *A*_6_ the starch-based SAP is the clear first choice. Once again, this fits with the actual historic motivation for exploring this option as part of the business’s overall R&D mix: starch-based SAP was a product with potentially interesting environmental credentials but was also clearly understood to be at an early stage of technology readiness, and so a high level of technical risk and uncertainty in whether a business model could be identified were tolerable, at least in the short term. 

Finally, returning to the analogy with a phase diagram, Figure 10 and Figure 11 display what a physical chemist would recognise as a triple-point, where three regions of first-ranking alternative meet at a single set of weights. A decision made at or near such a point would potentially be rather *non*-robust, with any one of several options liable to be judged best with just a small adjustment in the weightings in one direction or another. The more extreme weighting scenarios we have explored are unlikely to be generally applied in practice, nevertheless they provide a useful illustration that, in principle, decision outcomes *may* display sensitivity to weighting, and so conducting sensitivity analysis is recommended best practice.

### Knowledge capture

It should be emphasised that the narrative comments exemplified in Table 4 above and found in full in the companion data set form a vital part of the knowledge capture and documentation aspects of a *facilitated* decision-making process. The benefits of capturing this commentary in making the decision-making process explicit and transparent to affected stakeholders are clear, however adhering to this discipline also offers a potential for broader learning.

Innovation is a risky business and understanding *why* projects succeed or fail has already been the subject of typological research interest ( Crater & Lievense, 2018; McNulty, 1998). In this context, a coherent and thoroughgoing narrative may be considered as key *meta*-data, providing vital context on the decision, why it was arrived at, how, when and by whom. By tracking the further progress and ultimate success or failure of a sufficient body of innovation projects evaluated by MCDA, there is the potential to create a catalogue of exemplars from which *leading* indicators for successful projects might be derived, allowing the danger signs of likely ‘white elephant’ or dead-end projects to be spotted as early as possible in future. 

## Conclusions

We have demonstrated the use of a newly proposed two-level hierarchical framework for assessing relative sustainability amongst a set of alternatives from the earliest stages of process or product development. The FESSA framework has been applied successfully to a historic, real-world example using a spreadsheet-based implementation of the CURE decision-support model to support the underlying calculations. The decision framework identifies five independent top-level criteria as important indicators of process sustainability. Each top-level criterion is comprised of six to nine sub-criteria which proved in use to be both relevant and substantially independent of one another, thus allowing meaningful scores to be applied readily across a wide range of distinct process alternatives.

CURE provides a rapid method for numerical handling of complex decisions involving uncertainty that is computationally light, conceptually easy to grasp, and more discriminating between options than its predecessor MARE. It is an approximate method, but this is tolerable for many working purposes. Due to input variable scaling, the method can cope with a mixture of qualitative and quantitative scores as necessary, allowing decision-makers to express their assessment in convenient terms. This also makes the method suitable for progressive use as part of a stage-gate management process, where the volume and quality of data on the alternatives tends to increase, and the uncertainty to reduce, with the number of stages passed. 

Although the decision reached in the case study was robust, the importance of sensitivity analysis has been demonstrated. Getting the weights right is just as important as agreeing on the criteria and scores. Societal, and hence business, priorities may change with time and a valid historic decision may be viewed differently at a different point in time or in a different context. Those embarking upon decision-making activities should factor in time to think carefully about the weights applied before they attempt to score, and about the sensitivity of their conclusions to them afterwards.

In addition to the core duty of gathering data input, our proposed methodology encourages knowledge capture in the form of commentary about the alternatives, the weights applied in the decision set-up stage and how the input scores provided were arrived at. As such it provides a practical means for facilitating a group-based decision-making exercise and reporting its outcomes so that the results are reproducible and auditable. From the perspective of building a broader knowledge-based economy, if a sufficiently large body of consistently catalogued examples could be collected along with the subsequent project history, then these aspects might enable analysis to identify *leading* indicators for successful projects and allow early warning of white elephants.

## Ethics and consent

Ethical approval and consent were not required.

## Data Availability

Zenodo Digital Repository: Companion dataset to superabsorbent polymer manufacture case study described in paper “A framework for early-stage sustainability assessment of innovation projects enabled by weighted sum multi-criteria decision analysis in the presence of uncertainty”. https://zenodo.org/doi/10.5281/zenodo.10792826 ( Henderson & Peeling, 2024) The project contains the following underlying data CURE Companion Data Set.xlsx Data are available under the terms of the Creative Commons Attribution 4.0 International license.
