# Supplementary material for: A framework for early-stage sustainability assessment of innovation projects enabled by weighted sum multi-criteria decision analysis in the presence of uncertainty
Source: Open Res Eur. 2024 Aug 5;4:162. [Version 1] doi: 10.12688/openreseurope.18195.1 (PMC11459121; doi:10.12688/openreseurope.18195.1)
Supplement: Peer review history

# Reviewer report: Reviewer response for version 1

**DOI:** 10.21956/openreseurope.19665.r43076

**Published:** 2024-10-07

**Authors:** Syed Hammad Mian

**Affiliations:** 1 King Saud University, Riyadh, Saudi Arabia

First, I want to thank the authors, for their effort and hard work. The authors of this study have established a framework for multi-criteria decision analysis using the weighted-sum approach to assess sustainability in the presence of uncertainty.

 It is an interesting and excellent work. However, I request the authors to answer the following queries and make the necessary modifications.

1. What do you mean by uncertainty? What aspects are causing uncertainty in the decision-making process?
2. Given that the method is spreadsheet-based, it appears that as the number of alternatives and criteria rises, it may become more intricate and challenging to manage. Please discuss.
3. Adding weights to criterion or sub-criteria introduces a great deal of subjectivity into the decision-making process. How would you justify the allocation of weights in your methodology?
4. It gets more difficult or perplexing to provide weights to the criteria or sub-criteria as the number of criteria and sub-criteria increases. What are your opinions?
5. What distinguishes your method from fuzzy AHP, which likewise makes use of trapezoidal or triangular membership functions?
6. If there are limitations to your proposed approach, could you please outline them and explain how they might be addressed?
7. Please elaborate more on the future direction. How to expand on this work in the future?
8. Could you please list the assumptions that you considered for your proposed methodology?
9. A major obstacle to the use of MCDM techniques is the need for thorough and reliable gathering of data. In what way might it be made efficient and effective?

Is the rationale for developing the new method (or application) clearly explained?

Yes

Is the description of the method technically sound?

Yes

Are the conclusions about the method and its performance adequately supported by the findings presented in the article?

Yes

If any results are presented, are all the source data underlying the results available to ensure full reproducibility?

Yes

Are sufficient details provided to allow replication of the method development and its use by others?

Yes

Reviewer Expertise:

Multi-criteria Decision Making Analysis; Optimization; 3D Printing; Metrology; Manufacturing Processes; Reverse Engineering; Meta-Heuristics.

I confirm that I have read this submission and believe that I have an appropriate level of expertise to confirm that it is of an acceptable scientific standard.

# Reviewer report: Reviewer response for version 1

**DOI:** 10.21956/openreseurope.19665.r43470

**Published:** 2024-09-10

**Authors:** Soumava Boral

**Affiliations:** 1 University of Leeds, Leeds, England, UK

The article proposes a framework for early-stage sustainability assessment of innovation projects enabled by MCDM methods in the presence of uncertainty. The article needs to address the following issues before final acceptance:

1. Refer to Page No. 4, Introduction section: “We have therefore … early-stage process development” – what do you mean by ‘standard but flexible framework of criteria’? Could you please elaborate this statement further. ****I suggest elaborating this statement.
2. Next statement – “The FESSA…. Is designed for utility.” – What type of utility? Explain please.
3. Same paragraph, Introduction section – what do you mean by ‘hard data’? Need reference which explains the meaning of ‘hard data’.
4. Introduction section, 2 ^nd^ paragraph – “The thinking behind …real business cases” – Just wondering, how the environmental impacts imply the sustainability? Both terms are not same at all.
5. Refer to Page No. 6 of 19, 2 ^nd^ column, 2 ^nd^ paragraph – “In strict statistical applications … working purpose” – Could you please provide reference in support of your statement? In some statistical calculations, we consider sample size 80 as sufficiently large.
6. In the same paragraph, last line – “Provided that neither… misleading results.” – Suppose a particular criterion among n criteria has a weight of 0.69, and alternative value is high enough – then how could you solve this issue, either through MARE or through CURE?
7. The decision matrix, number of experts participated in this study, the biases of decision-makers, the knowledge levels of the participants were not taken into consideration. Even considered, it is not shown in the data source presented at the end.
8. The sensitivity analysis is not robust. There are other MCDA methods based on the probabilistic inputs, how could you compare your method with them?
9. The whole concept relies on Equation (6). I am just wondering, how your decision-making model could counter the fuzzy based approach of the MARE version? Let us assume, decision-scores follow a triangular fuzzy number, then what are the advantage of using Eq. (6), when more established fuzzy numbers are already available in the literature?
10. I also don’t agree with some of the criteria authors considered in their study. Let us consider the circularity (C37), which is far more complex, and itself considers multiple sub-criteria. How this criterion can be assessed by a simple term ‘circularity’ (assuming multiple experts provided their judgment)?

Is the rationale for developing the new method (or application) clearly explained?

Partly

Is the description of the method technically sound?

Partly

Are the conclusions about the method and its performance adequately supported by the findings presented in the article?

Partly

If any results are presented, are all the source data underlying the results available to ensure full reproducibility?

No

Are sufficient details provided to allow replication of the method development and its use by others?

No

Reviewer Expertise:

Circular economy, multi-crietria decision analysis (MCDA), Operations Management, Fuzzy logic, System dynamics, agent-based modeliing, machine learning, data analytics.

I confirm that I have read this submission and believe that I have an appropriate level of expertise to confirm that it is of an acceptable scientific standard, however I have significant reservations, as outlined above.

# Reviewer report: Reviewer response for version 1

**DOI:** 10.21956/openreseurope.19665.r43468

**Published:** 2024-08-29

**Authors:** Shankha Shubhra Goswami

**Affiliations:** 1 Abacus Institute of Engineering and Management, Hooghly, West Bengal, India

**Review comments**

- While the article mentions the improved discrimination of the CURE WSM-MCDA method over the MARE method, a more detailed comparative analysis would be beneficial. Providing quantitative comparisons or performance metrics could help in better understanding the advantages of the proposed method.
- The two-level hierarchical structure of the FESSA framework, combined with the new WSM-MCDA method, may introduce complexity. The article would benefit from a discussion on the usability of this framework for practitioners who may not have extensive expertise in multi-criteria decision analysis.
- Although the CURE method addresses uncertainty, the article could expand on how different types of uncertainty (e.g., epistemic vs. aleatory) are managed within the framework. Clarifying the scope and limitations of the approach in handling uncertainty would provide a clearer understanding of its applicability.
- The case study from the specialty polymer industry is strength, but the article would be stronger if it included a discussion on the generalizability of the framework and method across different industries or types of innovation projects. This would broaden the potential impact of the research.
- The article could benefit from a more thorough validation of the proposed framework and method, possibly including sensitivity analysis to assess the robustness of the outcomes to changes in input parameters or assumptions.
- The explanation of the FESSA framework and CURE WSM-MCDA method could be further clarified. Diagrams, flowcharts, or step-by-step guides might help in conveying the methodology more effectively to the readers.
- Future research could explore the application of the FESSA framework and CURE method in other sectors beyond manufacturing, such as services or software development, to test its versatility and adaptability.
- Investigating how the framework could be integrated with other decision-making tools or methods (e.g., life cycle assessment, cost-benefit analysis) could enhance its utility in broader contexts.
- Considering the dynamic nature of innovation processes, research into real-time or adaptive versions of the FESSA framework could provide valuable insights into how such tools can be used in rapidly changing environments.

**Decision: **Major revisions

Is the rationale for developing the new method (or application) clearly explained?

Yes

Is the description of the method technically sound?

Yes

Are the conclusions about the method and its performance adequately supported by the findings presented in the article?

Yes

If any results are presented, are all the source data underlying the results available to ensure full reproducibility?

Yes

Are sufficient details provided to allow replication of the method development and its use by others?

Partly

Reviewer Expertise:

Multi-criteria decision making (MCDM), Optimization techniques, Fuzzy decision-making, Fuzzy optimization, Industrial engineering, Management, Soft computing, Production management

I confirm that I have read this submission and believe that I have an appropriate level of expertise to confirm that it is of an acceptable scientific standard, however I have significant reservations, as outlined above.
